# Supplementary material for: Application of Multiple-Source Data Fusion for the Discrimination of Two Botanical Origins of Magnolia Officinalis Cortex Based on E-Nose Measurements, E-Tongue Measurements, and Chemical Analysis
Source: Molecules. 2022 Jun 17;27(12):3892. doi: 10.3390/molecules27123892 (PMC9229508; doi:10.3390/molecules27123892)
Supplement: Supplementary file 1 [file molecules-27-03892-s001.zip › molecules-1726795-supplementary.pdf]

Article

Application of Multiple-Source Data Fusion for the Discrimination of Two Botanical Origins of Magnolia Officinalis Cortex Based on E-Nose Measurements, E-Tongue Measurements, and Chemical Analysis

**Wenguang Jing [1], Xiaoliang Zhao [2], Minghua Li [1], Xiaowen Hu [1], Xianlong Cheng [1], Shuangcheng Ma [1,*]
and Feng Wei [1,*]**

[1] National Institutes for Food and Drug Control, Beijing 100050, China; jingwenguang@nifdc.org.cn (W.J.); bjlmmhh@163.com (M.L.); dreadless@126.com (X.H.); cxl@nifdc.org.cn (X.C.)
[2] Beijing Key Laboratory of Traditional Chinese Medicine Basic Research on Prevention and Treatment of Major Diseases, Experimental Research Center, China Academy of Chinese Medical Sciences, Beijing 100700, China; zhaoxiaoliang1218@aliyun.com
* Correspondence: masc@nifdc.org.cn (S.M.); weifeng@nifdc.org.cn (F.W.); Tel./Fax: +86-010-5385-2101(S.M.)

Table S1. Response values (*G*/*G0*) of the ten e-nose sensors for all samples (mean, duplicate=3) [1]

NO.	Origin	e-nose sensors									
		W1C	W5S	W3C	W6S	W5C	W1S	W1W	W2S	W2W	W3S
S1	JYHP	0.390	30.707	0.602	1.066	0.764	7.621	34.856	2.502	29.197	0.950
S2	JYHP	0.620	8.124	0.780	1.151	0.896	5.444	38.569	1.686	28.975	0.963
S3	JYHP	0.306	21.694	0.544	1.249	0.686	10.502	36.950	3.512	34.634	1.032
S4	JYHP	0.450	11.865	0.655	1.204	0.795	5.693	27.523	2.174	25.773	1.030
S5	JYHP	0.713	6.202	0.847	1.126	0.944	4.091	28.658	1.458	22.507	0.978
S6	JYHP	0.402	15.151	0.629	1.161	0.776	8.477	32.395	2.624	31.060	0.945
S7	JYHP	0.397	15.620	0.622	1.118	0.771	7.731	32.548	2.639	30.803	0.977
S8	JYHP	0.481	10.345	0.687	1.051	0.831	5.177	28.197	2.031	25.535	0.984
S9	JYHP	0.335	18.649	0.567	1.076	0.710	8.647	31.579	3.143	30.386	1.062
S10	JYHP	0.641	7.811	0.789	1.074	0.896	3.892	32.080	1.622	25.587	1.046
S11	JYHP	0.564	18.604	0.709	1.158	0.843	4.340	29.887	1.827	21.246	1.128
S12	JYHP	0.441	26.379	0.634	1.144	0.789	5.681	31.372	2.370	23.582	1.171
S13	JYHP	0.390	33.287	0.599	1.110	0.758	7.154	32.068	2.844	25.853	1.270
S14	JYHP	0.444	26.581	0.634	1.111	0.788	5.833	33.860	2.372	24.389	1.214
S15	JYHP	0.531	22.433	0.693	1.224	0.836	5.514	36.649	2.134	25.284	1.195
S16	JYHP	0.579	18.605	0.722	1.180	0.844	5.077	26.911	2.006	20.474	1.179
S17	JYHP	0.556	20.020	0.711	1.255	0.849	5.760	37.282	2.152	24.927	1.223
S18	JYHP	0.469	24.005	0.640	1.192	0.775	6.492	29.972	2.446	23.023	1.225
S19	JYHP	0.453	29.047	0.639	1.279	0.791	7.785	42.870	2.844	30.156	1.262
S20	JYHP	0.384	35.641	0.593	1.332	0.755	9.317	40.485	3.509	30.594	1.303

S21	JYHP	0.457	27.004	0.655	1.055	0.805	6.934	31.785	2.631	26.707	1.288
S22	JYHP	0.356	40.028	0.582	1.065	0.739	9.505	36.026	3.606	30.835	1.361
S23	JYHP	0.426	32.000	0.636	1.105	0.794	8.485	39.464	3.297	30.998	1.453
S24	JYHP	0.499	23.141	0.694	1.127	0.846	6.718	31.505	2.666	25.711	1.394
S25	JYHP	0.380	33.937	0.597	1.224	0.753	10.163	34.706	3.745	29.526	1.461
S26	JYHP	0.425	28.826	0.643	1.229	0.803	8.632	33.502	3.371	28.822	1.445
S27	JYHP	0.306	45.615	0.543	1.375	0.705	12.359	34.209	4.839	30.243	1.502
S28	JYHP	0.513	23.008	0.700	1.262	0.847	7.219	34.011	2.814	27.251	1.473
S29	JYHP	0.399	33.213	0.618	1.204	0.774	9.326	34.874	3.489	30.210	1.428
S30	JYHP	0.417	29.614	0.628	1.228	0.786	9.025	34.738	3.458	28.289	1.493
S31	AYHP	0.844	6.041	0.893	1.210	0.938	2.894	15.019	1.520	7.700	1.085
S32	AYHP	0.849	7.910	0.899	1.223	0.944	2.948	17.597	1.567	8.310	1.100
S33	AYHP	0.802	7.013	0.869	1.195	0.934	3.012	12.274	1.600	8.216	1.124
S34	AYHP	0.866	4.869	0.918	1.167	0.957	2.590	8.819	1.480	5.919	1.095
S35	AYHP	0.844	7.426	0.898	1.203	0.947	3.182	15.873	1.620	8.585	1.164
S36	AYHP	0.682	12.517	0.798	1.244	0.906	4.030	18.062	1.940	11.887	1.146
S37	AYHP	0.858	4.836	0.911	1.184	0.954	2.647	9.202	1.500	6.313	1.142
S38	AYHP	0.865	4.225	0.917	1.186	0.955	2.735	8.589	1.515	5.895	1.163
S39	AYHP	0.812	6.382	0.886	1.192	0.947	3.217	11.736	1.648	7.570	1.181
S40	AYHP	0.822	6.106	0.889	1.188	0.947	3.322	11.212	1.653	8.019	1.191
S41	AYHP	0.922	11.751	0.979	1.002	1.011	2.400	35.315	1.103	15.550	0.928
S42	AYHP	0.791	19.472	0.891	0.983	0.973	3.086	37.013	1.263	20.325	0.926
S43	AYHP	0.949	10.774	0.999	0.948	1.023	2.155	29.635	1.067	13.989	0.978
S44	AYHP	0.983	8.729	1.027	0.898	1.036	1.797	24.114	0.982	11.696	0.922
S45	AYHP	0.805	18.500	0.897	0.926	0.974	3.052	33.974	1.245	19.376	0.990
S46	AYHP	0.996	10.520	1.021	0.869	1.031	1.812	28.071	0.893	13.045	0.991
S47	AYHP	0.823	20.568	0.898	0.952	0.973	2.906	36.477	1.111	20.155	1.002
S48	AYHP	0.980	13.449	1.007	0.998	1.026	2.214	33.572	0.959	15.179	1.002
S49	AYHP	1.061	4.299	1.066	0.947	1.043	1.448	12.628	0.828	6.361	0.937
S50	AYHP	1.012	9.728	1.027	1.060	1.031	2.080	26.878	0.928	12.459	0.989
S51	AYHP	0.877	13.515	0.935	1.078	0.985	3.291	29.817	1.367	13.026	1.201
S52	AYHP	0.849	15.840	0.914	1.120	0.976	3.727	31.981	1.487	15.980	1.241
S53	AYHP	0.784	18.534	0.873	1.127	0.960	4.133	31.920	1.619	17.842	1.254
S54	AYHP	0.832	19.499	0.905	1.119	0.975	3.892	35.483	1.548	17.925	1.242
S55	AYHP	0.894	11.457	0.950	1.095	0.993	3.028	23.338	1.332	11.741	1.193
S56	AYHP	0.757	19.092	0.861	1.142	0.958	4.067	29.484	1.593	17.166	1.234
S57	AYHP	0.855	15.319	0.921	1.158	0.983	3.745	30.204	1.504	15.073	1.287
S58	AYHP	0.862	16.660	0.926	1.199	0.984	4.053	31.941	1.566	15.174	1.327
S59	AYHP	0.728	20.351	0.843	1.225	0.949	4.693	28.371	1.762	17.731	1.286
S60	AYHP	0.932	5.731	0.980	1.206	1.002	2.681	11.970	1.298	6.681	1.221

1 S1~S30 belongs to JYHP and S31~60 belongs to AYHP.

Table S2. Response values of e-tongue sensors for all samples (mean, n=3, mV)[1].

NO.	Origin	E-tongue sensor							
		Sourness	Bitterness	Astringency	Aftertaste- B	Aftertaste- A	Umami	Richness	Saltiness
S1	JYHP	-24.60	16.37	7.84	5.35	0.36	4.43	0.63	-16.38
S2	JYHP	-25.35	15.80	7.70	4.91	0.39	4.90	0.67	-15.76
S3	JYHP	-25.28	17.43	8.21	5.8	0.58	4.47	0.68	-16.46
S4	JYHP	-25.61	18.2	8.05	6.04	0.62	4.60	0.71	-16.18
S5	JYHP	-23.64	13.23	7.79	2.94	0.18	4.20	0.60	-16.85
S6	JYHP	-23.63	16.33	9.39	5.43	0.6	3.72	0.75	-17.48
S7	JYHP	-24.66	17.04	8.14	5.75	0.49	4.63	0.66	-16.5
S8	JYHP	-25.55	16.77	8.45	5.46	0.62	4.59	0.70	-16.65
S9	JYHP	-26.67	19.23	9.33	6.66	0.76	4.47	0.72	-17.09
S10	JYHP	-23.24	15.33	8.41	4.55	0.37	4.14	0.67	-17.1
S11	JYHP	-25.07	15.05	8.67	3.75	0.20	5.61	0.01	-15.97
S12	JYHP	-24.42	16.76	8.76	5.16	0.38	5.13	0.08	-16.34
S13	JYHP	-26.35	18.05	10.47	4.83	0.38	4.45	0.06	-17.08
S14	JYHP	-23.97	14.44	8.6	3.83	0.23	5.20	-0.01	-16.23
S15	JYHP	-24.24	16.12	8.54	4.72	0.27	5.12	0.08	-16.23
S16	JYHP	-25.13	16.81	8.81	4.99	0.32	5.36	0.07	-16.23
S17	JYHP	-24.15	14.97	8.71	3.95	0.22	5.18	0.03	-16.38
S18	JYHP	-24.84	17.54	8.91	5.89	0.51	5.28	0.06	-16.35
S19	JYHP	-23.88	15.23	7.86	4.58	0.28	5.38	0.02	-15.92
S20	JYHP	-23.49	15.97	8.17	4.88	0.28	5.16	0.02	-16.24
S21	JYHP	-25.14	17.87	8.09	5.74	0.36	5.45	0.26	-15.91
S22	JYHP	-25.75	17.03	8.16	5.52	0.53	5.68	0.24	-15.78
S23	JYHP	-22.12	14.75	7.75	4.82	0.32	4.79	0.24	-16.37
S24	JYHP	-23.84	15.58	8.26	4.71	0.36	4.96	0.24	-16.29
S25	JYHP	-24.94	16.42	7.43	5.54	0.46	5.82	0.24	-15.51
S26	JYHP	-25.03	16.67	8.10	5.57	0.39	5.33	0.30	-15.79
S27	JYHP	-25.03	16.21	8.04	4.46	0.30	5.47	0.25	-16.01
S28	JYHP	-25.59	15.46	7.51	4.63	0.31	5.96	0.19	-15.65
S29	JYHP	-25.64	16.04	8.39	4.56	0.33	5.61	0.21	-16.19
S30	JYHP	-23.94	16.36	7.67	4.82	0.27	5.44	0.2	-16.20
S31	AYHP	-28.01	12.46	5.56	2.38	0.05	8.37	0.18	-13.11
S32	AYHP	-26.37	13.41	4.01	3.52	0.13	8.55	0.21	-12.4
S33	AYHP	-28.54	13.01	6.70	1.99	0.06	8.23	0.14	-14.42
S34	AYHP	-27.69	12.96	5.92	2.40	0.06	8.38	0.15	-13.66
S35	AYHP	-28.59	14.66	6.14	3.35	0.16	8.50	0.19	-13.61
S36	AYHP	-30.67	23.78	7.9	6.89	0.32	9.06	0.22	-14.59
S37	AYHP	-27.92	13.35	5.76	2.35	0.07	7.74	0.19	-14.67
S38	AYHP	-31.6	14.77	5.33	2.56	0.04	10.51	0.07	-11.51
S39	AYHP	-31.38	14.58	7.38	2.65	0.12	9.76	0.13	-13.49
S40	AYHP	-28.02	16.26	6.98	3.97	0.26	7.85	0.19	-14.72
S41	AYHP	-23.9	12.56	4.76	3.53	0.27	6.78	0.01	-13.57

S42	AYHP	-24.65	19.42	5.36	6.47	0.36	6.78	0.00	-13.9
S43	AYHP	-23.92	19.86	4.12	6.79	0.25	6.98	0.06	-13.16
S44	AYHP	-22.94	21.03	4.25	8.18	0.3	6.76	0.12	-13.48
S45	AYHP	-24.05	15.8	5.91	4.69	0.34	6.44	0.02	-14.58
S46	AYHP	-23.81	18.2	6.50	4.62	0.17	6.24	0.03	-14.84
S47	AYHP	-24.1	15.76	6.48	4.88	0.41	6.48	0.01	-14.78
S48	AYHP	-23.28	18.62	6.24	5.02	0.22	6.45	0.03	-14.97
S49	AYHP	-25.39	13.74	5.85	3.35	0.25	7.12	-0.05	-13.8
S50	AYHP	-23.96	12.35	4.35	3.27	0.26	7.14	-0.03	-13.14
S51	AYHP	-24.72	10.06	6.75	0.96	0.08	6.43	-0.07	-14.73
S52	AYHP	-25	13.70	6.03	3.82	0.27	6.94	-0.01	-13.92
S53	AYHP	-24.09	13.08	8.41	2.68	0.22	5.39	-0.02	-16.32
S54	AYHP	-24.78	14.17	5.82	3.65	0.25	6.95	-0.06	-14.23
S55	AYHP	-24.35	13.39	4.92	3.44	0.24	7.17	-0.07	-13.33
S56	AYHP	-24.76	16.63	7.05	4.75	0.38	6.50	-0.02	-15.09
S57	AYHP	-23.99	12.94	6.21	3.03	0.24	6.50	-0.06	-14.28
S58	AYHP	-23.70	13.62	6.72	3.34	0.24	6.43	-0.02	-15.02
S59	AYHP	-24.98	15.53	8.13	4.31	0.43	6.30	-0.08	-15.60
S60	AYHP	-28.17	12.02	5.57	1.73	0.11	8.16	-0.13	-12.98

1 S1~S30 belongs to JYHP and S31~60 belongs to AYHP.

Table S3. Results of multi-component quantitative analysis (mean, n=2, mg/g) and proportionate features [1]

NO.	Syringin	Magnoflorine	Mag B#	Mag A#	Honokiol	Magnolol	Pipm#	Magnocurarine	β-eudesmol	THM	PBA	PHM
S1	4.292	1.744	44.494	6.690	17.137	30.837	10.628	2.047	.120	47.974	6.651	.556
S2	4.406	1.535	21.241	4.345	17.073	14.409	4.291	1.356	.000	31.482	4.889	1.185
S3	2.575	1.393	18.549	4.473	44.203	39.667	14.438	1.790	1.320	83.870	4.147	1.114
S4	1.542	1.011	11.590	4.550	37.826	29.862	9.110	1.568	.380	67.688	2.547	1.267
S5	3.150	2.070	29.160	3.060	16.270	17.540	5.390	1.750	.000	33.810	9.529	.928
S6	2.640	1.050	27.950	8.860	25.350	23.930	6.140	1.370	.810	49.280	3.155	1.059
S7	1.906	1.250	13.466	3.274	23.925	27.164	7.580	1.477	.000	51.089	4.113	.881
S8	2.493	1.256	20.711	3.110	29.678	21.248	4.712	1.914	.230	50.926	6.659	1.397
S9	1.979	1.009	8.455	2.794	40.267	38.866	9.140	1.309	.980	79.133	3.026	1.036
S10	3.274	1.393	24.718	4.143	15.111	17.640	2.926	2.092	1.460	32.751	5.966	.857
S11	1.930	1.000	15.900	3.470	13.980	13.310	3.300	1.090	.050	27.290	4.582	1.050
S12	3.080	1.805	27.726	4.500	33.139	29.110	9.740	1.628	.550	62.249	6.161	1.138
S13	3.883	1.065	21.176	4.128	27.920	22.306	9.350	1.299	.710	50.226	5.130	1.252
S14	1.630	1.390	15.360	4.060	32.750	30.200	15.430	1.870	.760	62.950	3.783	1.084
S15	2.834	1.156	17.674	3.738	21.409	19.010	6.363	1.373	.710	40.419	4.728	1.126
S16	3.828	1.501	54.275	12.326	30.015	22.629	7.657	3.121	1.160	52.644	4.403	1.326
S17	3.138	2.040	3.521	2.510	19.769	17.518	5.670	2.588	.010	37.287	1.403	1.128
S18	2.582	1.047	24.306	3.954	45.706	53.858	13.364	1.048	.260	99.564	6.147	.849
S19	3.268	1.646	27.036	4.513	23.815	25.902	7.396	1.624	.810	49.717	5.991	.919
S20	4.134	1.763	38.359	6.089	29.214	41.750	11.166	2.139	1.210	70.964	6.300	.700
S21	3.295	1.031	12.825	1.764	22.691	22.508	7.335	1.175	.530	45.199	7.270	1.008
S22	3.937	1.057	14.440	6.552	37.634	31.742	12.607	1.672	.000	69.376	2.204	1.186
S23	2.151	1.401	15.493	2.828	26.091	30.536	6.860	1.255	.700	56.627	5.478	.854
S24	1.815	1.070	12.585	3.094	24.631	17.140	6.159	1.494	.200	41.771	4.068	1.437

S25	2.201	1.206	9.989	5.061	32.554	34.357	8.874	1.445	.530	66.911	1.974	.948
S26	1.980	1.250	27.620	4.810	28.640	23.080	6.770	1.490	1.700	51.720	5.742	1.241
S27	2.920	2.370	7.050	15.030	7.990	14.900	.000	3.930	.000	22.890	.469	.536
S28	3.733	1.297	35.840	4.716	21.849	18.247	4.831	1.557	.680	40.096	7.600	1.197
S29	3.324	1.375	31.615	5.190	25.727	23.060	11.924	2.002	.860	48.787	6.092	1.116
S30	2.125	1.624	18.879	13.247	25.090	23.296	6.652	1.974	.480	48.386	1.425	1.077
S31	2.542	2.349	6.361	8.863	5.431	9.765	.969	3.919	.390	15.196	.718	.556
S32	3.789	2.265	9.816	14.421	9.885	17.921	1.006	4.341	1.200	27.806	.681	.552
S33	2.473	1.381	4.329	4.494	4.235	8.248	1.391	1.858	.140	12.483	.963	.513
S34	3.962	1.184	5.349	11.865	4.515	8.710	.764	3.013	.100	13.225	.451	.518
S35	2.712	2.358	4.450	8.547	7.934	14.461	.783	3.004	.590	22.395	.521	.549
S36	3.568	1.608	12.257	8.505	15.400	23.517	2.732	2.106	.570	38.917	1.441	.655
S37	3.328	1.760	5.369	18.264	8.267	11.358	.904	3.053	.850	19.625	.294	.728
S38	4.701	1.544	5.231	11.665	4.039	5.594	.893	3.656	.110	9.633	.448	.722
S39	3.593	2.100	10.659	12.582	8.404	11.731	1.461	.982	.040	20.135	.847	.716
S40	2.195	2.186	3.372	10.029	9.319	19.772	.802	2.225	.620	29.091	.336	.471
S41	2.994	2.109	6.319	11.400	8.919	16.529	.755	3.544	.750	25.448	.554	.540
S42	3.811	1.719	13.388	8.801	11.866	18.842	2.340	2.145	.490	30.708	1.521	.630
S43	3.263	2.617	6.412	11.178	7.934	14.525	.833	3.913	.840	22.459	.574	.546
S44	3.323	2.594	7.244	11.282	9.094	16.773	.834	4.289	.960	25.867	.642	.542
S45	3.895	1.690	16.240	9.397	11.745	18.874	2.618	2.222	.120	30.619	1.728	.622
S46	2.537	1.114	6.148	5.743	4.458	5.737	.841	1.549	.520	10.195	1.071	.777
S47	4.045	1.716	15.665	8.838	12.900	20.416	2.675	2.319	2.580	33.316	1.772	.632
S48	2.858	1.037	7.477	5.467	5.308	6.850	.790	1.598	.220	12.158	1.368	.775
S49	4.205	1.170	13.473	14.306	5.743	11.546	.909	2.833	.270	17.289	.942	.497
S50	3.651	2.371	8.264	13.661	8.742	16.489	.797	4.235	1.010	25.231	.605	.530

S51	3.310	1.620	13.090	10.940	10.700	17.010	2.190	1.880	.000	27.710	1.197	.629
S52	3.219	2.657	6.399	12.286	10.714	19.914	.871	3.436	1.820	30.628	.521	.538
S53	2.170	1.190	14.070	10.520	4.330	8.370	.000	2.540	.410	12.700	1.337	.517
S54	2.756	1.251	9.733	14.363	8.181	17.520	1.001	2.603	1.330	25.701	.678	.467
S55	3.120	2.531	6.481	13.961	8.463	16.066	.829	4.176	.980	24.529	.464	.527
S56	4.052	1.798	12.835	11.361	12.827	19.863	2.335	2.300	.450	32.690	1.130	.646
S57	3.202	2.693	6.770	10.510	8.482	15.744	.831	4.134	.760	24.226	.644	.539
S58	3.016	2.315	6.808	10.472	9.476	17.404	.800	3.411	.920	26.880	.650	.544
S59	3.360	1.613	13.468	10.929	13.733	21.180	2.424	2.154	.550	34.913	1.232	.648
S60	2.429	1.142	13.656	2.974	19.805	17.230	3.038	1.232	.750	37.035	4.592	1.149

[1] S1~S30 belongs to JYHP and S31~60 belongs to AYHP. *n.d., not detected. # Mag B=Magnoloside B; Mag A=Magnoloside A; Pipm =Piperitylmagnolol.
